# Detection of Total Hip Replacement Loosening Based on Structure-Borne Sound: Influence of the Position of the Sensor on the Hip Stem

**DOI:** 10.3390/s24144594

**Published:** 2024-07-16

**Authors:** Nico Schumacher, Franziska Geiger, Sascha Spors, Rainer Bader, Christian Haubelt, Daniel Kluess

**Affiliations:** 1 Applied Microelectronics and Computer Engineering, University of Rostock, 18059 Rostock, Germany; christian.haubelt@uni-rostock.de; 2 Department of Orthopaedics, Rostock University Medical Center, 18057 Rostock, Germany; rainer.bader@med.uni-rostock.de (R.B.); daniel.kluess@med.uni-rostock.de (D.K.); 3 Institute of Communications Engineering, University of Rostock, 18059 Rostock, Germany; sascha.spors@uni-rostock.de

**Keywords:** total hip replacement, implant loosening, vibration analysis, resonance frequency analysis, modal analysis

## Abstract

Accurate detection of implant loosening is crucial for early intervention in total hip replacements, but current imaging methods lack sensitivity and specificity. Vibration methods, already successful in dentistry, represent a promising approach. In order to detect loosening of the total hip replacement, excitation and measurement should be performed intracorporeally to minimize the influence of soft tissue on damping of the signals. However, only implants with a single sensor intracorporeally integrated into the implant for detecting vibrations have been presented in the literature. Considering different mode shapes, the sensor’s position on the implant is assumed to influence the signals. In the work at hand, the influence of the position of the sensor on the recording of the vibrations on the implant was investigated. For this purpose, a simplified test setup was created with a titanium rod implanted in a cylinder of artificial cancellous bone. Mechanical stimulation via an exciter attached to the rod was recorded by three accelerometers at varying positions along the titanium rod. Three states of peri-implant loosening within the bone stock were simulated by extracting the bone material around the titanium rod, and different markers were analyzed to distinguish between these states of loosening. In addition, a modal analysis was performed using the finite element method to analyze the mode shapes. Distinct differences in the signals recorded by the acceleration sensors within defects highlight the influence of sensor position on mode detection and natural frequencies. Thus, using multiple sensors could be advantageous in accurately detecting all modes and determining the implant loosening state more precisely.

## 1. Introduction

Due to good clinical results, the increasing demand by patients aged 64 years or younger, and the aging of the population, the number of hip joint arthroplasty procedures will increase in the future [1,2]. As a result, the number of revision surgeries will also increase, especially for younger patients [3,4,5]. There is a trend towards cementless fixation, especially in younger patients [5,6]. In Germany, uncemented fixation is the standard method for primary hip arthroplasty, accounting for 77 percent of cases [7,8]. Implant loosening is one of the most common reasons for follow-up procedures [7,9]. Imaging methods, such as radiology, are generally used as the standard procedure for diagnosing implant loosening, as they are usually available in the clinical setting and are more cost-effective and convenient to use [10]. Unfortunately, these methods only allow for examinations in a clinical environment, while detection as early as possible would be favorable without regular appointments in a clinic. Additionally, Temmermann et al. [11] have shown in their study that the sensitivity and specificity of imaging-based techniques only slightly exceed 80%. However, accurate prediction of implant loosening is necessary in order to recognize it at an early stage and take timely intervention to prevent the destruction of bone material. Consequently, extensive research has been performed to develop novel methods for the non-invasive detection of loosening, which could possibly mitigate some of the shortcomings of imaging-based techniques. Surveys of alternative methods can be found in Hosseini et al. [10], Cachão et al. [12], and Vickers et al. [13]. Among the various methods, vibrometric approaches have demonstrated promising results. Georgiou et al. [14] concluded that vibrational analysis has a 20% higher sensitivity in detecting the loosening of total hip implants compared to radiographs, which here shows a sensitivity of 60%. Furthermore, resonance frequency analysis is already considered a reliable technique for measuring the stability of dental implants [15]. Vibration analysis relies on measuring the vibration characteristics of the bone implant system, which is expected to change according to the loosening state.

Following Cachão et al. [12], the approaches in this field can be roughly divided into four categories according to their excitation and measurement principles. These categories are depicted in Table 1. For vibrational analyses, it is essential to apply excitation at all frequencies over the entire spectrum, which should be observed. This was either achieved using transient impulses, for example, through hammer strikes [16], or through harmonic excitation using an exciter or shaker [17,18,19]. Acceleration sensors are usually used to record the vibrations. In most cases, the vibration-based methods rely on extracorporeal mechanical excitation and extracorporeally recorded vibrations [12]. The issue with extracorporeal mechanical excitation or measurement is that the excitation and the signal can be dampened due to soft tissue interactions so that little to no signal is received [17,20,21,22]. In addition, the measurements are not reproducible due to various factors such as positioning deviations and changes in body composition [20]. However, the reproducibility of the measurements is important, as in vibration analyses, the measured data must be compared with the existing individual data of the fixed implant in order to detect loosening. Moreover, utilizing an extracorporeal exciter for excitation can result in discomfort and pain, rendering this principle inappropriate for certain patients [14]. In order to solve these problems, instrumented total hip endoprostheses were introduced to allow intracorporeal excitation and/or measurement directly at the implant.

Ruther et al. [20] provided a new technology for internal excitation. Here, a magnetic oscillator integrated into the implant is made to vibrate via an externally attached electromagnetic coil, and the implant is mechanically stimulated by the impact. However, the vibrations were recorded extracorporeally by an acceleration sensor attached to the skin. This technology has shown good results [23,24,25,26]. In these studies, however, easily distinguishable defects were examined, so that the extracorporeally recorded measurement signal was sufficient. In order to be able to detect even small differences in loosening in the future, the damping effect of the soft tissue on the measurement signal should be minimized. Puers et al. [19], Marschner et al. [17], and Sauer et al. [18] embedded an accelerometer directly into the implant to enable more accurate intracorporeal measurements. For that, the accelerometer was integrated into the implant head [18,19] or at the distal end of the femoral stem [17].

To the best of the authors’ knowledge, there is no study on how to best position the sensor on the stem to detect implant loosening. However, the mode shapes of numerical simulations [27,28] indicate that the position of the measurement directly on the implant may be relevant for the recording of natural frequencies.

Therefore, the aim of this study is to investigate the mechanical excitation of the vibrations and the recording of the signals directly on the implant in various bone defects. For this purpose, the signals of several accelerometers placed at different positions along the implant were analyzed. In addition, the Finite Element (FE) method was used to determine the bending modes and natural frequencies of the respective models.

## 2. Materials and Methods

### 2.1. Experimental Setup

**Figure 1:**
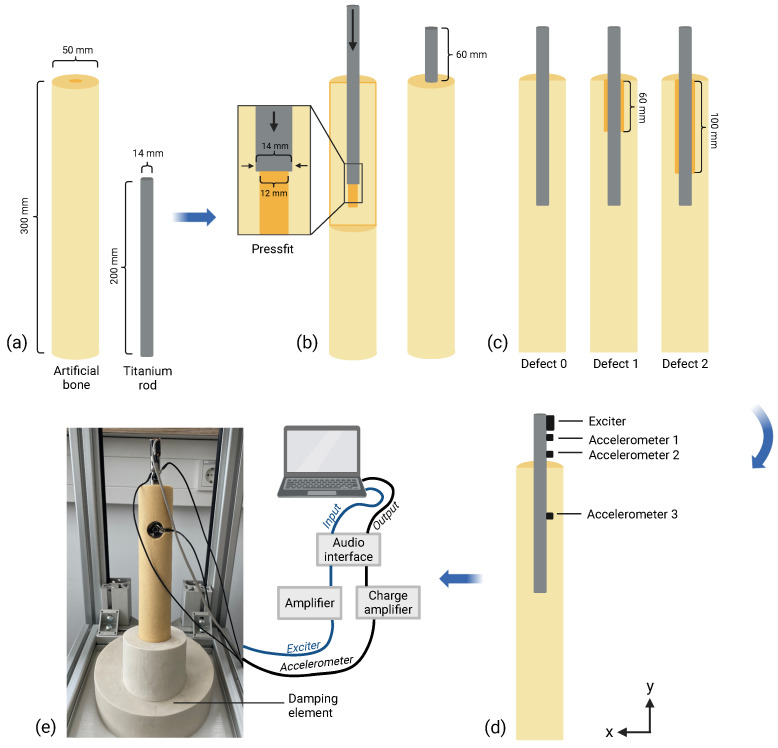
Construction of the experimental test setup: (**a**) materials for the model used and their dimensions, (**b**) insertion of the titanium rod into the artificial bone, (**c**) investigated peri-implant defects, (**d**) attachment of the exciter and the accelerometers, (**e**) measurement test setup (created with Biorender.com).

Instead of using a commercial total hip replacement stem, a rod made of titanium alloy (Ti6AI4V) was used as a model for the hip implant. This was implanted in a cylinder of closed-cell polyurethane foam (density ρ=10 pcf, Sawbones, Vashon, WA, USA) to model the cancellous part of the bone. This architecture considerably simplifies the assembly of the accelerometers and the exciter. The experimental setup can be seen in Figure 1.

The total length of the titanium rod was 200 mm, of which 140 mm was inside the sawbone. The 60 mm sticking out was based on the length of the vertical offset (vertical line from center of the head to the lesser trochantor). The titanium rod was fixed by a pressfit. This was achieved by drilling a hole with a diameter of 12 mm in the sawbone and then hammering the titanium rod with a diameter of 14 mm into the sawbone. The sawbone itself, with a length of 300 mm and a diameter of 50 mm, was embedded in a rubber damping element with a depth of 75 mm to decouple it from the environment. A total of three different loosening models were examined. Defect 0 was an intact bone model, i.e., the titanium rod was in direct contact with the cancellous bone along the whole length of the embedding. For bone defects 1 and 2, peri-implant defects were inserted. For this purpose, additional bone material with a diameter of 16 mm was removed proximally before implantation of the rod so that after insertion of the rod, there was a defect of 1 mm around it. This was carried out to a depth of 60 mm for defect 1 and 100 mm for defect 2.

An exciter (BCE-1, Dayton Audio, Springboro, OH, USA) was screwed onto the proximal end of the titanium rod so that the excitation took place in the x-direction, as shown in Figure 1d. It had a small form factor and allowed for excitation in the frequency range between 300 Hz and 19 kHz [29]. It was driven by an amplifier (CS-PA1 MK II, Dynavox, Iffezheim, Germany), which obtained the playback signal from the Evo 8 audio interface (Audient Limited, Herriard, Hampshire, UK).

Additionally, three one-dimensional accelerometers (KS95B100, Metra Mess- und Frequenztechnik, Radebeul, Germany) were attached to the titanium rod with grub screws so that the direction of the recording was in the x-direction. These were located at a distance of 30 mm (accelerometer 1), 50 mm (accelerometer 2), and 120 mm (accelerometer 3) from the proximal end of the titanium rod, i.e., accelerometer 1 was located directly under the exciter, accelerometer 2 was directly at the proximal end of the sawbone, and accelerometer 3 was approximately in the middle of the implanted part of the titanium rod. These acceleration sensors were connected to a charge amplifier (M68D3, Mess- und Frequenztechnik, Radebeul, Germany), from which the output was fed into the audio interface. This allowed for synchronized playback and recording of the signals. All the data were sampled at 48 kHz. For each defect model, three consecutive recordings were recorded without any alterations to the setup before changing to another defect model. The analog electrical part of the setup, including the exciter, the amplifiers, and the audio interface, as well as the gain settings, were not altered between consecutive measurements of different defects.

In order to exclude variations induced by modifications during the switch of the defect models that were not related to the defect size, additional experiments were conducted to investigate possible variations induced by the conversion of the experimental setup. For this purpose, the position of the sawbone in the damping element was varied by rotating the sawbone by 0, 10, and 20 degrees, whereas 0 degrees was defined as the standard position. Additionally, the fixation of the sawbone was examined by pulling the sawbone out of the damping element by 0, 5, and 10 mm, with 0 mm used as the standard fixation. Furthermore, three measurements for removing and reattaching the exciter and three measurements for removing and reattaching the accelerometers were performed.

### 2.2. Signal Processing

To measure the frequency response for each of the accelerometers, an exponential sine sweep was used. Compared to other approaches, an exponential sine sweep is a simple yet deterministic way to obtain the frequency response, which has already been applied to the vibrational analysis of hip prosthesis [30,31]. The parameters of the exponential sine sweep are displayed in Table 2. The exponential sweep was conducted from 50 Hz to 20 kHz, which covers the whole usable bandwidth provided by the exciter and all relevant frequency ranges demanded by Qi et al. [28]. Additionally, one second of silence was added before and after each sine sweep [30]. After recording the signal, the measured signals were zero padded to convert the cyclic convolution into a linear convolution and transformed into the frequency domain using the Fast Fourier Transform (FFT). Then, the frequency response $H_{i}\left(\mu \right)$ for each accelerometer was obtained via spectral division:

$$H_{i}\left(\mu \right)=\frac{Y_{i}\left(\mu \right)}{X(\mu )}$$

where X(μ) is the spectrum of the exponential sine sweep, which was played back using the exciter, $Y_{i}\left(\mu \right)$ is the spectrum of the recorded signal for accelerometer *i*, and μ denotes the discrete frequency. The obtained frequency response is then averaged over the three recordings for each defect. The spectral division was also used to obtain the transfer function between two accelerometers. This transfer function combines the frequency information from two sensors. In our experiments, accelerometer 1, positioned directly beneath the exciter, could serve as the reference signal for the excitation. In contrast, accelerometer 3, located farthest from the exciter, could be viewed as the system’s output signal. By looking at the transfer function between accelerometers 1 and 3, we can obtain the system’s response at the location of accelerometer 3, which might be less dependent on variations in the excitation. This could be particularly useful if the excitation is not fixed and can vary over several recordings. Similar to before, the recorded signals for the corresponding accelerometer were zero-padded and transformed into the frequency domain to obtain the transfer function. The transfer function $H_{31}\left(\mu \right)$ between accelerometer 3 and 1 is obtained as follows:

$$H_{31}\left(\mu \right)=\frac{Y_{3}\left(\mu \right)}{Y_{1}\left(\mu \right)}$$

where $Y_{3}\left(\mu \right)$ and $Y_{1}\left(\mu \right)$ are the spectra of accelerometers 3 and 1, and μ denotes the discrete frequency. Similar to before, the obtained transfer function is averaged over the three recordings for each defect.

The degree of nonlinear behavior was presented by Rosenstein et al. [32] as another measure for detecting aseptic loosening. To analyze the degree of nonlinearity, a pure sine was played back over the exciter for 10 s at a frequency of 450 Hz in the unaltered experimental setup. This frequency was chosen because it corresponds to the first eigenmode for all defects in the measurements. Similar to the sine sweep measurements, 1 s of silence was added before and after each pure sine wave playback. Nonlinearities should appear in the recordings as modulations on the base frequency sine wave in the time domain or as additional peaks in the frequency domain at multiples of the excitation frequency.

### 2.3. Modal Analysis

To examine the bending modes and natural frequencies of the investigated models, a modal analysis was performed using FE modeling using Abaqus 2022 (Dassault Systèmes Simulia Corp., Providence, RI, USA). The geometries of the sawbone and titanium rod were constructed as shown in Figure 1. The materials of the cancellous bone and titanium alloy were assumed to be linear elastic, and the corresponding densities were defined. The exact material properties can be found in Table 3.

The parts were meshed with hexahedral elements with a quadratic function (C3D20) with an average element edge length of 3 mm. The model of defect 0 consisted of 22,389 elements, defect 1 consisted of 21,644 elements, and defect 2 consisted of 21,164 elements. The independence of the results from the mesh size was determined via a convergence analysis.

**Figure 2:**
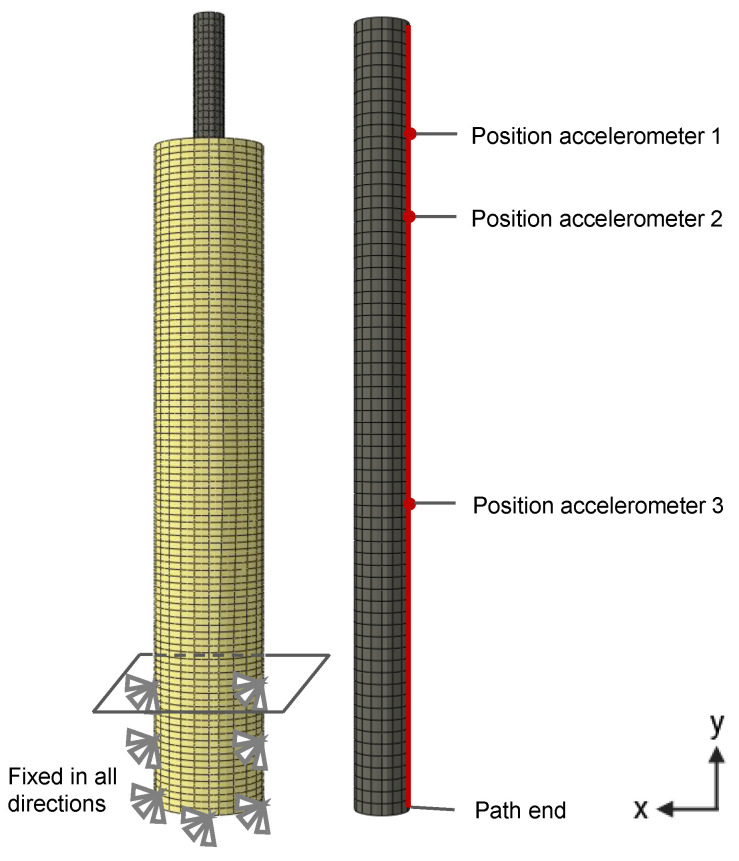
Construction of the FE model for modal analysis and definition of the titanium rod path used for the analysis.

The cancellous bone was fixed at the distal end, with a length of 75 mm in all directions. The normal contact between the polished titanium rod and the cancellous bone was assumed to be hard, and a friction coefficient of 0.08 [36] was defined for the tangential contact. To simulate the press fit as shown in Figure 1, an interference fit was used with the titanium rod as the main surface and the cancellous bone as the secondary surface. The final FE model is shown in Figure 2. The modes and natural frequencies of the models were then analyzed in a frequency range of 0–3000 Hz. Furthermore, it was examined how well the accelerometers might be able to detect the bending modes depending on the attached position. For this purpose, the bending mode shape deformation amounts in the recording direction (x-direction) along the path of the stem defined in Figure 2 were investigated. The bending mode shape deformation was displacement-normalized, which means that the peak amplitude was normalized to a value of 1.

## 3. Results

As described in Section 2.1, several measurements were carried out to test the reproducibility of the experimental setup. Regarding the variations for consecutive removal and reattaching of the exciter, the variations between the frequency responses obtained from the experiments were smaller than 1 dB. The same held true for consecutive removal and reattaching of the accelerometers, where the variations in the peak heights did not exceed 3 dB. Looking at the variations induced by pulling the sawbone out of the rubber feet, the qualitative shape of the frequency response stayed identical. For the measurement where the sawbone was pulled out 10 mm, minor differences of less than 5 dB were visible in the frequency range of 500 Hz to 1.5 kHz in all three accelerometers, whereas for the experiment with the sawbone pulled out 5 mm, the variations were even smaller. For rotating the sawbone inside the rubber feet, the variations between the measurements did not exceed 3 dB across the whole recorded spectrum.

In all experiments for the sensitivity analysis, the frequencies of the peaks in the frequency response stayed nearly identical. For frequencies exceeding 10 kHz, more variations emerged across repeated experiments, especially with each successive remount of the exciter. Therefore, the following comparison was only conducted up to a frequency of 10 kHz, where the experimental setup proved to be highly repeatable, and possible influencing factors in the measurement system could be excluded for each modeled defect. Additionally, diagrams up to a frequency of 3 kHz are shown in this section to better investigate the lower-frequency behavior, especially in the sensitive band proposed by Qi et al. [28].

### 3.1. Frequency Response Analysis

**Figure 3:**
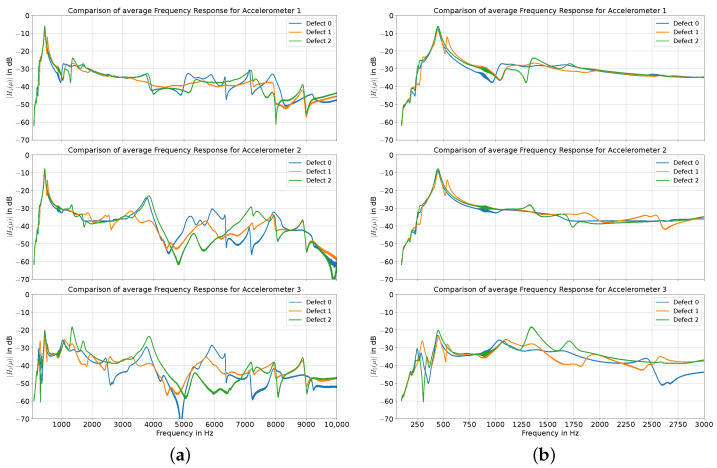
(**a**) Frequency responses for accelerometers 1–3 for defect 0 (blue), defect 1 (orange), and defect 2 (green) within the frequency range of 0 to 10,000 Hz and (**b**) 0 to 3000 Hz averaged over 3 recordings.

Figure 3 shows the frequency responses for all the accelerometers and all three defects. The x-axis displays the frequency, and the y-axis shows the magnitude of the frequency response over three recordings. Each diagram represents the recordings of one of the accelerometers. The colors represent the three types of defects considered in our experiments. A clear mode is discernible at approximately 450 Hz, which is consistent for all defects and for all accelerometers.

Unlike accelerometers 1 and 2, the frequency response of accelerometer 3 shows another mode at around 350 Hz for all defects. An additional mode is visible in all accelerometers at around 550 Hz only for defect 1. Accelerometers 1 and 3 show a mode between 1000 and 1100 Hz for all defects. This is the first mode where a frequency shift can be seen between the defects. Contrary to expectations, the frequency of the mode is higher when the titanium rod is less tightly secured, leading to a rightward shift in the modal behavior. Above 1100 Hz until 3000 Hz, defect 0 only shows one additional mode at around 2450 Hz, which is only visible for accelerometer 3. The recordings for defect 1 show two peaks at around 1900 and 2550 Hz, which stand out for accelerometers 2 and 3 but are only marginally visible for accelerometer 1. Similarly, for defect 2, two modes are visible at 1350 and 1700 Hz. Compared to accelerometers 2 and 3, the frequency response of accelerometer 1 is relatively flat for all defects at frequencies between 2000 Hz and 4000 Hz. Looking at the frequency responses above 3000 Hz, an upward frequency shift between defect 2 and defect 0 for the peak at approximately 3900 Hz can be observed. Generally, the frequency responses between the modeled defects differ quantitatively by up to 20 dB and qualitatively by the shape and position of the modes. Nevertheless, at some points, similarities between the modeled defects become apparent; for example, at 8000 and 8900 Hz for defects 1 and 2, where the mode at 8900 Hz has a nearly identical shape, amplitude, and frequency for defects 1 and 2 for all the accelerometers.

### 3.2. Transfer Function between Accelerometers

**Figure 4:**
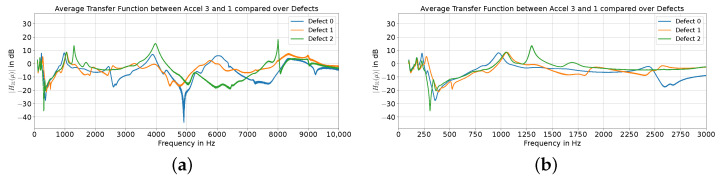
(**a**) Transfer functions between accelerometer 3 and 1 for defect 0 (blue), defect 1 (orange), and defect 2 (green) within the frequency range of 0 to 10,000 Hz and (**b**) 0 to 3000 Hz averaged over 3 recordings.

As described in Section 2.2, one approach for combining the data from multiple accelerometers as a classification feature could be to compute the transfer function in between. Figure 4 displays the magnitudes of the computed transfer functions between accelerometers 1 and 3 for all three defects averaged over three recordings. Especially in the lower-frequency spectrum, the differences between the defects are better visible in the transfer function compared to the frequency responses for each accelerometer. As before, modal behavior can be seen across the whole frequency range, and the defects can be clearly distinguished. At higher frequencies (>5000 Hz), the variations between the defects are smaller compared to the frequency response variations described before.

### 3.3. Analysis of Nonlinearities

**Figure 5:**
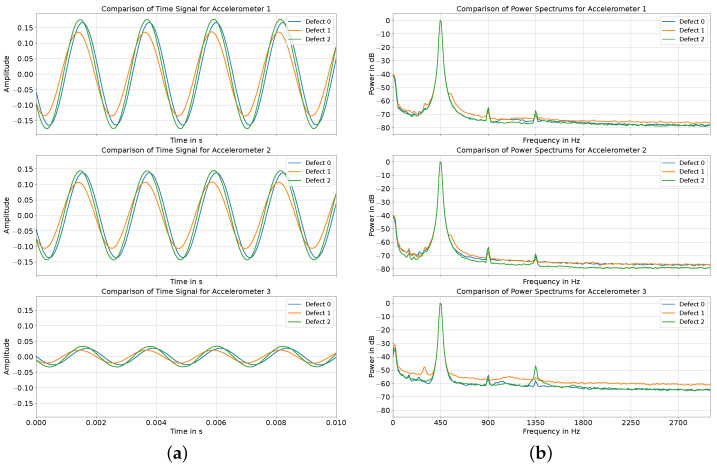
(**a**) Time signal from all 3 accelerometers recorded for a sine wave at 450 Hz for all 3 defects. (**b**) Power spectra obtained from all 3 accelerometers for the sine wave at 450 Hz for all 3 defects.

To analyze nonlinear behavior, a pure sine wave was played through the exciter. As detailed in Section 2.2, this sine wave, with a frequency of 450 Hz corresponding to the first and highest mode identified in the frequency response analysis, was played for 10 s. Similar to [32], the degree of nonlinearity was visually compared in the time and frequency domains between the defects. Figure 5a displays the time domain signals for all defects for all the accelerometers. For each defect, the sinusoidal excitation is visible for every accelerometer. Although no changes were made in the settings of the playback devices between the experiments, the amplitude of the oscillation for defect 1 was marginally smaller. At the same time, there were only minor differences in the maximum amplitude between defects 0 and 2. Comparing the maximum amplitude of the sine wave between the accelerometers, the amplitude of accelerometer 3 was significantly smaller for all the defects compared to the signals recorded for accelerometers 1 and 2. Between accelerometers 1 and 2, only a minor decrease in amplitude was observable. Regardless of the defect, no nonlinear behavior in the form of higher-frequency modulations was visible in the time domain signals.

In Figure 5b, the power spectra of the recordings for all the defects across all the accelerometers are presented. The frequency is marked on the x-axis, and the y-axis shows the power of a particular frequency bin. The power spectrum was obtained using the Welch method, with a Hann window and a window size of 4096. All the spectra were normalized to the maximum power of the respective signal. Across all the accelerometers and defects, the excitation signal at 450 Hz is clearly discernible in the power spectrums and marks the highest peak. The reduced signal amplitude of the sine waves in accelerometer 3 also becomes evident in the spectra for all the defects due to its lower signal-to-noise ratio (SNR) compared to accelerometers 1 and 2. Overall, the power spectrums show that the recorded signals contain nonlinear components that are not visible in Figure 5a due to the small amplitude of the harmonic components. Looking at the first harmonic at 900 Hz, no significant differences in the power are visible between the defects. This holds true for all the accelerometers. For the second harmonic at 1350 Hz, the maximum power between the defects remained nearly identical, with only minor variations. However, examining the spectra for accelerometer 3 reveals an increase in power for the second harmonic for defect 2. At frequencies above 900 Hz, no higher-order harmonics are visible in the power spectra for any of the accelerometers and across all defects.

### 3.4. Modal Analysis

**Figure 6:**
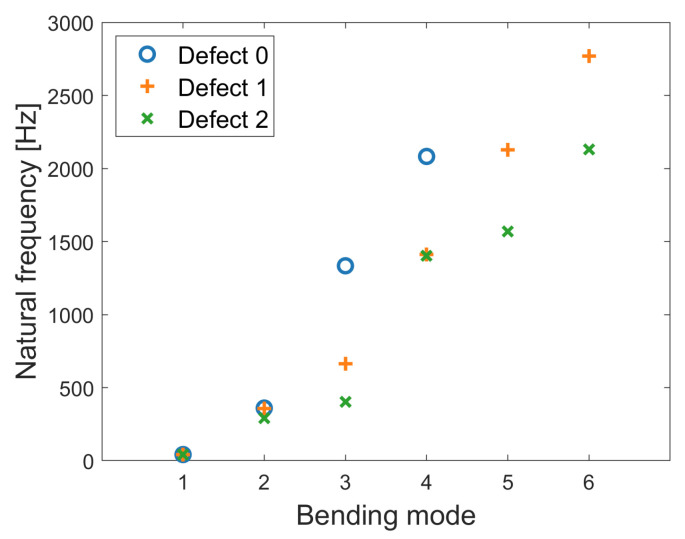
Natural frequencies of the bending modes depending on the different defects.

The results of the natural frequencies of the bending modes of the respective defects are shown in Figure 6. The first two natural frequencies of the bending modes hardly differ between the defects. From mode 3 onwards, the natural frequencies decrease as the defect size increases. An outlier can be seen in bending mode 4, where defects 1 and 2 show a similar natural frequency of approximately 1400 Hz. In the considered frequency range of 0–3000 Hz, defect 0 has four natural frequencies, and the other two defects have six.

**Figure 7:**
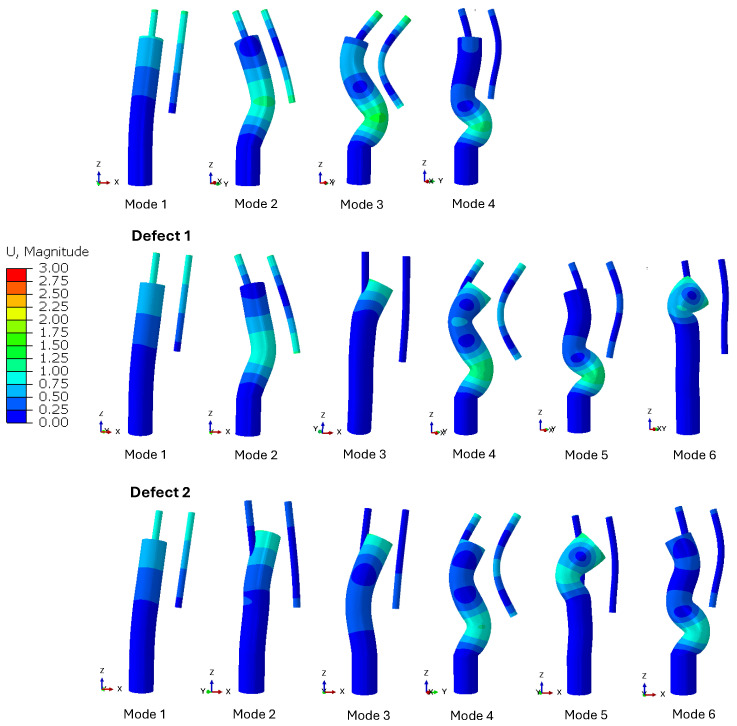
Bending mode shapes of the different defects normalized by displacement and scaled to a deformation factor of 30.

The corresponding bending mode shapes of the respective defects normalized to displacement with a deformation factor of 30 are shown in Figure 7. The shapes of the first bending modes of the defects are similar. From mode 2 onwards, defect 2 has a different mode shape than the other two defects. From mode 3 onwards, the mode shapes of defects 0 and 1 also differ significantly. The shape of mode 3 of defect 0 is similar to the shape of mode 4 of defects 1 and 2. Furthermore, there are similarities in mode 4 of defect 0, mode 5 of defect 1, and mode 6 of defect 2. Looking at the natural frequencies of similar mode shapes in Figure 6, it can be seen that they also have similar frequencies.

**Figure 8:**
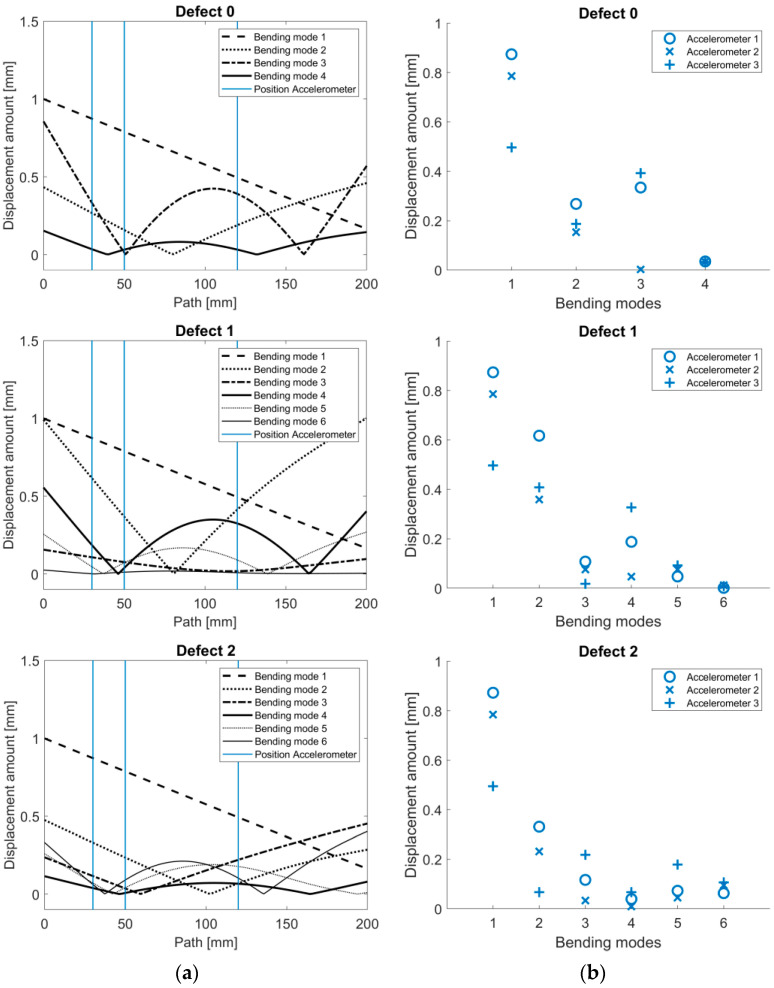
(**a**) Bending mode shape amount normalized to displacement along the titanium rod path and (**b**) at the position of the different accelerometers on the titanium rod for the bending modes of the different defects.

In Figure 8a, the displacement-normalized bending mode shape deformation amounts were plotted in the x-direction along the path of the stem for the different defects, with additional positions of the accelerometers included. Additionally, Figure 8b shows the bending mode shape amount normalized by the displacement at the position of the acceleration sensors for the respective bending modes of the defects. It can be seen that some of the accelerometers were located near a node, such as accelerometer 2 at bending mode 3 of defect 0 and at bending mode 4 of defect 2. Here, the deflection amplitude is close to zero, so these modes can hardly be detected, or not at all. For all the defects, bending modes 1 and 2 were best detected by accelerometer 1. For the higher bending modes, accelerometer 3 detected the modes better, e.g., mode 3 of defect 0, modes 4 and 5 of defect 1, and modes 3–6 of defect 2. The last mode could hardly be detected by any of the accelerometers.

## 4. Discussion

Overall, our experimental setup and measurement system showed a high repeatability throughout multiple experiments in the frequency range below 10 kHz. The more prominent differences caused by rotating and slightly pulling out the sawbone were significantly smaller than the variations between the defects. The differences in the experimental setup caused by the modifications were usually situated in higher frequencies. Nevertheless, during the initial development of the experimental setup, variations throughout repeated experiments were observed, which were likely caused by minor unwanted variations in the assembly process of the experimental setup. This suggests that minor variations in the mechanical setup can exert a significant influence on the measured signals, especially in the higher-frequency range, which is crucial to consider for real-world implementation of the system later on. When designing such an implant system, one has to make sure that the mechanical variations, which are not related to the loosening state of the implant, remain as small as possible during the lifetime of the implant in order to not influence the consecutive measurements.

The frequency response of the experiment and natural frequencies of the numerical modal analysis cannot be directly compared with each other, since a modal analysis generally does not take into account any structural dampening effects or the excitation signal produced by the exciter. A full modal analysis with accelerometer sensors at suitable points along the rod was not possible, since the sawbone and the rod would have been damaged too much due to the placement of the accelerometers. Hence, a numerical modal analysis was performed to obtain essential information about the mode shapes and the deflection at the positions of the accelerometers. However, it must be taken into account that the FE model is a simplification and is therefore only an approximation of reality.

The results of the modal analysis indicate that the placement has an influence on the magnitude of the natural frequencies recorded. Especially in the first modes, the displacements are high for all the accelerometers; as the frequencies increase, they typically tend to decrease. But, within a mode, there are differences in the displacement that are dependent on the positions of the accelerometers, which means that the placement of the accelerometer significantly impacts the potential detection of modal behavior. The results of the modal analysis are also reflected in the experimental results. The experiments show obvious differences in the magnitude of the detected natural frequencies between the accelerometers at the corresponding defects. One example is the nearly flat frequency response of accelerometer 2 for defect 0 in Figure 3b at frequencies higher than the first natural frequency, which is supported by the results of the displacement-normalized mode shape amount in Figure 8. Generally, the modal analysis for our setup in Figure 8 indicated that accelerometers 1 and 2 should experience a larger displacement for the first 2–3 modes, whereas the deflection for accelerometer 3 should be larger for higher-frequency modes compared to accelerometers 1 and 2. These predictions can also be observed in the experimental results, where the frequency responses for accelerometers 1 and 2 show larger magnitudes for the first mode, which were probably also amplified by the closer proximity to the excitation source. Similar to the modal analysis results, the measured frequency responses indicate that accelerometer 3 is more suited to detect modes above 1200 Hz due to the larger magnitudes observed, which can be seen for the mode at around 2400 Hz for defect 0, which was only detectable using accelerometer 3. In accordance with the simulation results, the frequencies of the modes below 1000 Hz only marginally differed between the defects for all the accelerometers, except for some variations around 300 Hz, which were, however, mostly outside of the linear frequency range of the exciter. The modal analysis backs up these results and is in line with the simulatory results reported by Qi et al. [28], who categorized the frequency spectrum as follows: the range from 500 to 1500 Hz was deemed to be less sensitive, while frequencies above 1500 Hz were considered to be sensitive, and those above 2500 Hz were classified as highly sensitive for the differentiation of defects.

The model analysis predicts that similar mode shapes will have similar natural frequencies between the defects, with the frequencies increasing slightly as the size of the defect increases, as can be seen in Figure 6 and Figure 8. Looking at the experimental results in Figure 3, one can also observe an increase in the natural frequency at around 1000 Hz for defects 1 and 2 for accelerometers 1 and 3. Looking at the natural frequencies independent of the mode shapes, there is a decrease in the natural frequencies, which is also shown in related literature [17,18,28].

As proposed, combining the information of multiple sensors could be beneficial to better distinguish between the defects, for example, by computing the transfer function between accelerometer 1, which is closest to the exciter and could be considered as the excitation reference signal, and accelerometer 3, which could be regarded as the output signal of the system. Similar to the frequency response analysis, large differences between the defects are visible across the analyzed frequency spectrum, which would again allow for a simple classification for our limited sample set. The concept of embedding one sensor close to the excitation source could provide another benefit. By having a reference of the excitation signal available, it could be possible to make the evaluation independent of the variations in the excitation. Assuming that the excitation is sufficient across the whole frequency band, variations in the excitation system could be resolved by digital signal processing, which might be especially helpful if the excitation could vary across multiple trials.

As an alternative method for detecting the loosening states, the degree of nonlinearity for each defect was investigated. Nonlinear behavior was not visible in the time domain signals displayed in Figure 5a, whereas a slight increase in the power of the second harmonic at 1350 Hz became visible for defect 2 in accelerometer 2 in the frequency domain analysis in Figure 5b. This increase in harmonic distortions aligns with the results obtained by Rosenstein et al. [32] and Puers et al. [19]. However, their increase in harmonic signal components was significantly larger and also visible in the time domain signals [19,32]. Consequently, the degree of nonlinearity is likely not well-suited for the practical implementation of a detection system, as the differences in our experiments were only marginal. Furthermore, the excitation itself significantly influences the results, especially the excitation’s power. During our experiments, we noticed that overdriving the exciter also led to significant nonlinear components in the signal, especially around the first harmonic at 450 Hz. Nevertheless, placing an accelerometer close to the excitation source again proves advantageous. As this signal is closely related to the excitation signal, it would be straightforward to notice whether the nonlinearities likely originate from the system or the excitation source itself.

Overall, as our experimental setup is highly reproducible, a differentiation between the three modeled defects is easily possible by comparing the recorded frequency response to the three model frequency responses in the corresponding sensor. Also, the comparison of the transfer function between accelerometers 1 and 3 showed clear variations between the modeled defects, which would also enable the detection of loosening by visually comparing the transfer function obtained. The increase in nonlinear components for the loosened implant seems to be the least reliable means of detection, as the increase was only marginal. Also, other classification approaches could be used, like a shift in the resonance frequencies, used, for example, by Sauer et al. [18]. Nevertheless, our limited and simplified test setup is not suited for the evaluation of classification measurements, which would require, for example, a larger sample size and a more realistic representation of the real-life conditions.

In summary, both the experiments and the modal analysis point out that combining the information and data from multiple accelerometers is expected to allow for more reliable detection of the vibrational modes for each defect. It is expected that for more complex mechanical setups, like an actual implant with a shape differing from our cylindrical model, the position dependence of the accelerometer for recording multiple modes would be even higher. This further motivates the approach of placing multiple accelerometers at defined locations to improve the early detection of implant loosening.

A significant limitation of our investigations is the degree of abstraction of the experimental setup. Both the bone and the implant were represented by cylindrical structures. This leads to a simplified vibrational behavior compared to modeling the implant–bone system in more detail, like in [27,28]. The bending modes and frequency values will be different for a hip implant compared to a rod. Nevertheless, compared to studies with more complex models of femurs and implants, similar trends in the changes in frequencies in relation to the different defects are shown [27,28,37].

Furthermore, the artificial bone made out of polyurethane foam mimics only the cancellous bone. The cortical bone and soft tissue were neglected in this study. This leads to further deviations from the real system, because the surrounding soft tissue can impact the frequency behavior through dampening, reductions in peaks, and lower resonance frequencies, as shown by Makris et al. [21]. We performed an FE analysis using cortical bone, which showed that the cortical bone would also change the frequency values, but the frequency trend between the defects would be similar. Yousefsani et al. [31] also showed similar effects. In addition, the mode shape would change between defects, so the position of the accelerometers also plays a role here.

A limitation of our experimental setup is the accuracy of the excitation. The excitation signal for calculating the frequency response is assumed to be X(μ), which is equivalent to the theoretical signal generated by the computer. In general, this signal can deviate from the real excitation vibration signal produced by the exciter on the titanium rod. However, this does not limit the validity of our contribution. The signal-generating components of the experimental setup were not altered between experiments involving a comparison between the defects. This means that the measured frequency response can be seen as the concatenation of the constant frequency response of the signal-generating components and the frequency response of the vibrating implant. This implies that changes in the measured frequency responses in our experiments are purely caused by changes in the system’s mechanical part, which is exactly what we expected. A force sensor could be included in future experiments to measure the excitation and allow for a more sophisticated frequency response analysis.

Another limitation of our study is the limited number of samples investigated in our experiments. In total, only three models were analyzed. Nevertheless, our research was not focused on comparing markers regarding the detection accuracy for loosening but rather on promoting the benefits of using multiple accelerometers on one implant while also investigating the position dependence.

## 5. Conclusions

This study presented a novel sensor concept that could, in the future, improve hip implant loosening detection accuracy using structure-borne sound analysis. To the best of our knowledge, only single-accelerometer approaches have been proposed to detect the fixation state of an uncemented hip implant. Instead of relying on one accelerometer positioned at a single location on the implant, our proposal involves employing multiple accelerometers to measure vibrations at various points across the implant. In this work, it was shown that the position of the recording has an important influence on the detection of natural frequencies and modes. The information from several acceleration sensors could enhance the detection of small differences between defects. In the future, more experiments with more realistic models and variations in the models have to be conducted in order to further investigate the advantages of using multiple accelerometers for the detection of implant loosening. If the presented method enables detecting implant loosening early and more sensitively in the future, unnecessary revision operations could be avoided, or therapeutic measures could be applied earlier, e.g., electrical stimulation of the bone in the area of the defect, which could possibly even prevent revision of the implant. This could save considerable costs in the future. Moreover, the exposure to radiation due to X-ray examinations can be diminished using this approach.

## Figures and Tables

**Table 1:** Overview of the possible combinations of excitation (eE—external excitation; iE—internal excitation) and measurement (eM—external measurement; iM—internal measurement) for vibrational methods in the literature according to [12].

		Excitation	
		Extracorporeal	Intracorporeal
**Measurement**	**Extracorporeal**	Category eE eM	Category iE eM
	**Intracorporeal**	Category eE iM	Category iE iM

**Table 2:** Overview of the sine sweep parameters used throughout the experiments.

Parameter	Value
Start frequency	50 Hz
End frequency	20,000 Hz
Duration	10 s
Silence interval before and after sweep	1 s

**Table 3:** Material properties of the artificial bone and titanium rod used for the finite element model.

	Elastic Modulus [MPa]	Poisson’s Ratio [−]	Density [kg/m^3^]
Titanium rod [33,34]	110,000	0.316	4470
Sawbone [35]	86	0.3	160

## Data Availability

The data presented in this study are available from the corresponding authors upon request.
